# Application of associating liver partition and portal vein ligation for staged hepatectomy for initially unresectable hepatocellular carcinoma

**DOI:** 10.1186/s12893-022-01848-w

**Published:** 2022-11-24

**Authors:** Haoqi Chen, Xiaowen Wang, Wenfeng Zhu, Yang Li, Zhenyu Yu, Hua Li, Yang Yang, Shuguang Zhu, Xiaolong Chen, Genshu Wang

**Affiliations:** 1 Department of Hepatic Surgery, Liver Transplantation, The Third Affiliated Hospital of Sun Yat-Sen University, Guangzhou, China; 2 Guangdong Key Laboratory of Liver Disease Research, The Third Affiliated Hospital of Sun Yat-Sen University, Guangzhou, China

**Keywords:** ALPPS, TACE, FLR, Liver regeneration

## Abstract

**Objective:**

To evaluate the safety and efficacy of associating liver partition and portal vein ligation for staged hepatectomy (ALPPS) in the treatment of initially unresectable hepatitis B virus (HBV)-associated hepatocellular carcinoma (HCC) and to preliminarily explore the mechanism of rapid growth of the future liver remnant (FLR).

**Methods:**

Twenty-four patients with HBV-associated HCC who underwent ALPPS in our hospital from August 2014 to January 2021 were retrospectively studied. Propensity score matching was used to compare oncologic outcomes of patients treated with ALPPS and transarterial chemoembolization (TACE). The expression of YAP and JNK in liver tissue after two stages of ALPPS were detected.

**Results:**

The median standard liver volume (SLV) was 1471.4 ml. Before second stage of ALPPS, the median FLR increased by 74.4%, and the median FLR/SLV increased from 26.1 to 41.6%. Twenty-two patients (91.7%) received staged hepatectomy after a median interval of 15 (9–24) d. The total incidence of postoperative complications in ALPPS group was 54.5%, and of Clavien–Dindo ≥ IIIb postoperative complications (requiring surgical, endoscopic or radiological intervention under general anesthesia) was 9.1%. There was no significant difference in total complications between ALPPS group and TACE group, but there were lower rate of above grade III complications in the TACE group than that in the ALPPS group. The incidence of complications was lower in laparoscopic-ALPPS than that in open surgery. In ALPPS group, the 1-year, 2-year and 5-year overall survival rate were respectively 71.4%, 33.3% and 4.8%. Interval time was an independent risk factor associated with overall survival rate. There was no significant difference in overall survival rate between ALPPS group and TACE group. For advanced HCC (BCLC stage B and C), ALPPS group was not superior to TACE group in overall survival rate. The expression of YAP and p-JNK in the residual liver tissue after second stage procedure was higher than that after first stage procedure, and the co-expression of YAP and p-JNK was observed in the residual liver tissue.

**Conclusion:**

ALPPS is a safe and effective treatment for initially unresectable HBV-associated HCC. Laparoscopic technique might improve the effect of ALPPS. YAP and JNK pathway might take a role in rapid FLR increase in ALPPS procedure.

**Supplementary Information:**

The online version contains supplementary material available at 10.1186/s12893-022-01848-w.

## Introduction

Hepatocellular carcinoma (HCC) is one of the most common malignancy in China, accounting for 75–85% of liver cancer [1], which is associated to chronic hepatitis B infection (about 80%) [2, 3]. Surgery is the most effective treatment for HCC. Unfortunately, the resectability of HCC is only 20–30% [4]. One important factor limiting the HCC resectability is the insufficient future liver remnant (FLR) and post-hepatectomy liver failure. For HCC with inadequate FLR, transarterial chemoembolization (TACE) or TACE combined with targeted drugs and immunotherapy are currently the main treatments. However, TACE is a palliative treatment with a certain risk of tumor recurrence and progression. Additionally, the repetitive treatments are required and potentially cause severe liver injury [5]. Since the associating liver partition and portal vein ligation for staged hepatectomy (ALPPS) were accidentally created by Hans Schlitt [6] in Germany, it has brought hope to patients with initially unresectable HCC because of its ability to effectively increase FLR in a short period. But it has also been controversial due to the high postoperative complications and mortality rates. With the development of damage control and employment of minimally invasive techniques, the adverse effects of ALPPS have been gradually reduced, making it a safe and effective treatment for initially unresectable hepatitis B virus (HBV)-associated HCC. But its long-term effect needs to be investigated. Meanwhile, the mechanism by which FLR increases rapidly in ALPPS procedure remained unclear.

Yes-associated protein (YAP) is the core regulatory element of Hippo signaling pathway. YAP activated enters the nucleus and binds with the transcription factor TEAD to activate downstream genes, which plays an important role in regulating liver size. Recent studies [7–9] have demonstrated that that Hippo signaling is essential in the regeneration of multiple tissues and organs, such as the heart, liver, and skin. In the mouse model of partial resection, YAP is activated due to inhibition of MST1/2 and LAST1/2 after liver resection, which promotes cell proliferation, but its specific regulatory mechanism has not been clarified [10]. In addition, c-Jun N-terminal kinase (JNK) is an evolutionarily conserved mitogen-activated protein kinase, which is one of the important signaling pathways regulating cell proliferation, survival and death. Recent studies [11–13] have demonstrated that JNK-deficient mice show significantly impaired liver regeneration after 2/3 partial hepatectomy. For treatment of initially unresectable HBV-associated HCC, the underlying mechanism of YAP and JNK signaling pathways in ALPPS to accelerate liver regeneration is unknown.

The purpose of this study was to present our experience in treatment of initially unresectable HBV-associated HCC with ALPPS. The oncological outcomes of ALPPS and TACE were compared by propensity score matching (PSM). At the same time, we preliminarily explored the mechanism of rapid increase of FLR in ALPPS procedure by detecting the expression of YAP and JNK in liver tissue after two stages of ALPPS.

## Methods

All procedures of this study were in accordance with the Helsinki Declaration and the approval arrangement of the Ethics Committee of the Third Affiliated Hospital of Sun Yat-sen University, and all patients signed informed consent approved by the hospital ethics committee.

### Patients

Twenty-four consecutive patients with HCC who received ALPPS in our hospital from August 2014 to January 2021 were retrospectively analyzed. Patients included in this study were either positive for HBV surface antigen (HBsAg) or had detectable HBV DNA (viral markers used for diagnosis of HBV). All patients required routine preoperative magnetic resonance imaging (MRI) or computed tomography (CT) examination to exclude extrahepatic metastasis. Additionally, 330 patients with HBV-associated HCC who received TACE alone in our hospital from August 2014 to January 2021 were retrospectively analyzed. All patients were stratified according to the Barcelona Clinical Liver Cancer Staging System (BCLC) [14]. Postoperative complications were defined according to the Clavien–Dindo criteria [15].

### Assessment of liver volume

Before and after first stage ALPPS, the FLR was measured by three-dimensional reconstruction using the IQQA-Liver CT interpretation and analysis system (EDDA Technology Inc, Princeton, NJ), and the standard liver volume (SLV) was estimated by the Urata formula [16]. For patients with cirrhosis, preoperative FLR/SLV > 40% is considered safe. For patients with no evidence of cirrhosis, preoperative FLR/SLV > 30% is considered safe [17]. The future residual liver weight (FRLW) and FRLW/BW (body weight) were calculated. When FRLW/BW > 0.5%, the second stage of ALPPS could be performed safely [18]. The kinetic growth rate (KGR), which reflects the daily growth of FLR, was calculated.

### Liver reserve function

Liver function and coagulation indices were detected before and 1, 2, 3, 7, 9, 11 d after first stage of ALPPS, and 1, 3, 5, 7 d after the second stage of ALPPS. Preoperative liver function grade was required to be Child–Pugh Grade A or Grade B adjusted to Grade A. Additionally, patients with postoperative liver insufficiency were defined as having at least the following two outcome measures: serum total bilirubin (TBIL) > 60 μmol/l, a prothrombin time (PT) rate < 30% of the normal level, alteration of consciousness, and asterixis. The 15-min retention rate (R15) of indocyanine green (ICG) was measured by DDG-3300K, and R15 < 10% is needed for safe major hepatectomy [19].

### Surgical procedure

The ALPPS procedure is carried out as reported by de Santibañes [6]. The first stage procedure includes: exploring cavity to rule out any intraperitoneal extrahepatic metastases; resecting gallbladder; dissociating and transecting right branch of portal vein; spitting the right and left lobe of liver along with the right side of middle hepatic vein. The second stage procedure includes: transecting right branches of hepatic artery and bile duct; transecting the right hepatic vein close to Inferior vena cava; dissociating and removing the right lobe of liver. TACE procedure is carried out as previously described [20]. In our hospital, digital subtraction angiography was used to guide super-selective catheterization to the arterial branch of intrahepatic lesion, and oxaliplatin, 5-fluorouracil, epirubicin or mitomycin C mixed with 5–30 ml lipiodol was injected. Stage embolization was performed with microcatheters for small lesions.

### Immunohistochemical, immunofluorescence and Western Blotting

Liver tissues were sampled during the first and second stage procedure of ALPPS, fixed by 4% neutral paraformaldehyde, embedded in paraffin, then sectioned in 4 μm slice. Immunohistochemical staining of YAP, p-JNK were performed with anti-YAP (1/200, Cell Signaling Technology, USA), anti-p-JNK (1/200, Cell Signaling Technology). Bound antibodies were visualized with Dako REAL™ EnVision™ Detection System Peroxidase/DAB + kit, and slices were counterstained with hematoxylin. For immunofluorescence, bound secondary antibodies conjugated with FITC and Cy3 (1: 500, Beyotime) were performed at room temperature for 1 h along with DAPI (5 mg/ml, Beyotime). Images were captured for quantification with a Zeiss microscope and quantified with ImageJ software.

For Western Blotting, the collected fresh liver tissue is homogenized, cleaved and centrifuged to obtain liver tissue proteins. The blots were cut prior to hybridisation with antibodies during blotting. Immunoblotting was performed according to the manufacturer’s instructions using the following antibodies: anti-YAP, anti-JNK, anti-p-JNK, anti-GAPDH, and HRP-conjugated goat anti-rabbit IgG antibodies (all the antibodies were obtained from Cell Signaling Technology, USA).

### Follow-up

After discharge, patients were followed up monthly for the first 6 months and every 3 months thereafter.

### PSM analysis

PSM was used to compare oncologic outcomes of patients treated with ALPPS and TACE during the same period. PSM was used to reduce the confounding effect on measured covariates. ALPPS and TACE were matched 1:1 as closely as possible using the following variables: age, sex, α-fetoprotein (AFP), nutrition risk screening (NRS) score, MELD score, tumor number, tumor size, macroscopic vascular invasion, distant metastasis, lymphatic node metastasis, ascites, albumin (ALB). The primary endpoint was overall survival (OS), incidence of postoperative complications.

### Statistical analysis

Continuous variables with a normal distribution are represented as mean ± standard deviation, and nonnormally distributed data are represented as the median. When the variance hypothesis met normality and homogeneity, the continuous data were compared using the paired T-test. For categorical variables, the Chi-square test was used. Univariate and multivariate Cox regression analyses were used to test the prognostic factors related to OS. Survival was analyzed by Kaplan–Meier analysis and was compared with the log-rank test. All statistical analyses were performed using SPSS (Version 25.0, IBM, Chicago, USA) and GraphPad Prism (Version 8, San Diego, USA) statistical software.

## Result

### Patients

24 HBV-associated HCC patients who received ALPPS during the study period were all male. The median age was 47.5 y (range 30–77 y). The median maximum diameter of single tumor was 7.3 cm in 16 cases, and multiple tumors were 2.8 cm in 8 cases. There was major vascular invasion in 7 cases, including 1 case involving the right portal vein, 1 case involving the right hepatic vein, 2 cases involving the right portal vein and right hepatic vein, 2 cases involving the right hepatic vein and middle hepatic vein, and 1 case involving the right portal vein and middle hepatic vein. According to the staging system of BCLC, there were 5 cases in stage A, 10 cases in stage B and 9 cases in stage C (Table 1).

**Table 1:** Patients characteristics

Patients characteristics	Data
Age, median (range), y	47.5 (30–77)
Sex, male/female, n (%)	24/0 (100/0)
Single tumor (n = 16)	
Maximum tumor diameter, median (range), cm	7.3 (4.3–11.9)
Multiple tumor (n = 8)	
Maximum tumor diameter, median (range), cm	2.8 (2.5–11)
Vascular invasion, n (%)	
Right portal vein	1 (4.2)
Right portal vein + right hepatic vein	2 (8.3)
Right hepatic vein	1 (4.2)
Right portal vein + Middle hepatic vein	2 (8.3)
Right hepatic vein + Middle hepatic vein	1 (4.2)
Ascites, n (%)	
None	12 (50)
Small	10 (41.7)
Moderate	2 (8.3)
Portal hypertension, n (%)	
None	11 (46.8)
Yes	13 (54.2)
NRS score (2002)^*^, n (%)	
2	17 (70.8)
3	6 (25)
4	1 (4.2)
BCLC Classification, n (%)	
A (early)	5 (20.8)
B (intermediate)	10 (41.7)
C (advanced)	9 (37.5)

BCLC Classification, Barcelona Clinic Liver Cancer Classification

^*^NRS score (2002), Nutritional risk screening 2002

### Liver volume and liver reserve function

**Fig. 1:**
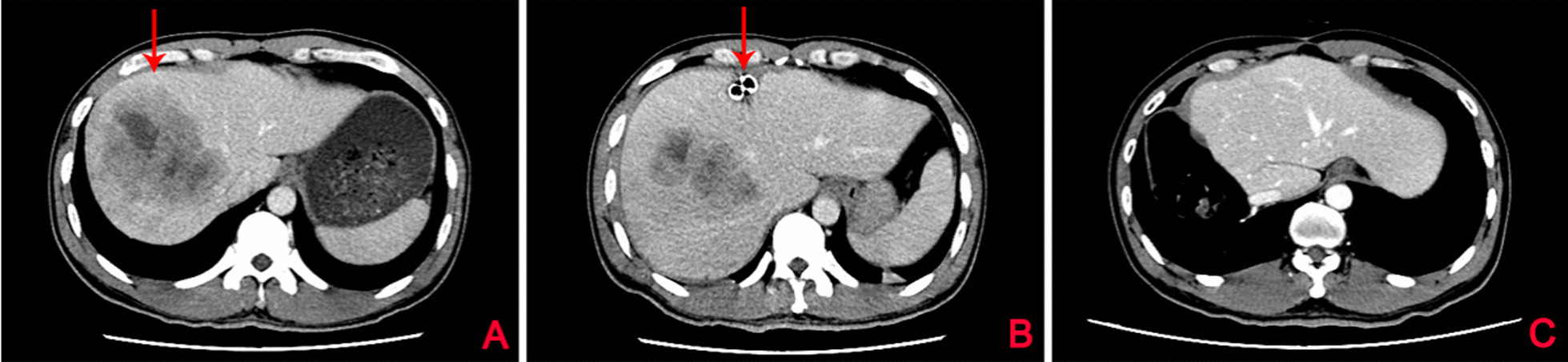
CT scan before and after ALPPS. **A** Patient’s preoperative CT scan. The red arrow pointed to the tumor, which was located in the right liver; **B** CT scan performed on the 3 d after the first stage of ALPPS. The red arrow indicated where the liver was dissected in the first stage procedure; **C** CT scan of the patient 3 months after the second stage of ALPPS

**Fig. 2:**
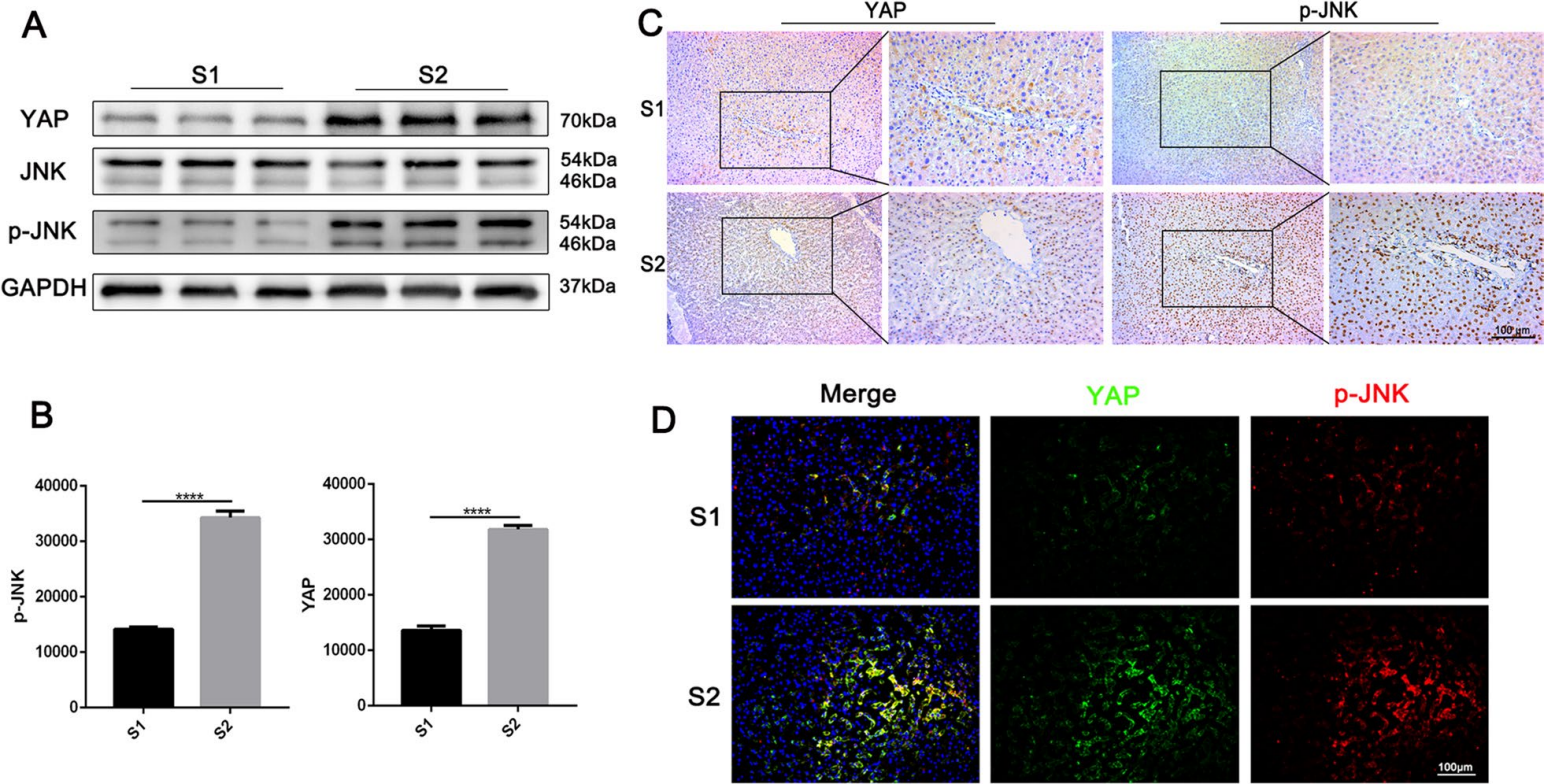
The expression of YAP and p-JNK in liver remnant tissue of two stages procedure. **A**, **B** Western blot-assisted YAP, JNK and p-JNK expression in liver remnant tissue of two stages procedure (p < 0.001 by Student’s t test); **C** Immunostaining for YAP and p-JNK in liver remnant tissue of two stages procedure (scale bar, 100 µm); **D** Double fluorescent staining for YAP (green), p-JNK (red) and DAPI (blue in liver remnant tissue of two stages procedure (scale bar, 100 µm)

**Fig. 3:**
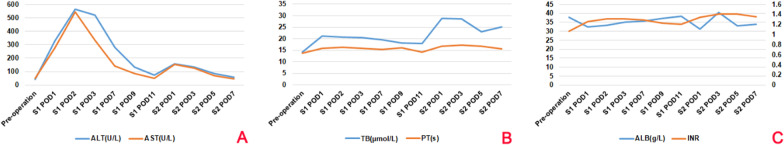
Changes in liver function after ALPPS procedures. **A** Y-axe represented ALT (blue) and AST (orange). After the first and second stage procedure, AST and ALT increased sharply, but decreased rapidly; **B** Y-axe represented TB (blue) and PT (orange). TB showed a marked increasing in the first day after the first and second stages procedure, but steadily fall after that. PT changed little after ALPPS. **C** Dual Y-axes represented ALB (blue) on the left Y-axis and INR (orange) on the right. ALB decreased slightly and increased gradually after the first stage procedure, and slightly fluctuated after the second stage procedure but was higher than 30 g/l. INR changed little after ALPPS; AST, aspartate aminotransferase; ALT, alanine aminotransferase; ALB, albumin; PT, prothrombin time; INR, international normalized ratio; TB, total bilirubin; S1, the first stage procedure; S2, the second stage procedure; POD, post-operative day

Before first and second stage procedure of ALPPS, the liver volume was measured by the IQQA-Liver CT interpretation and analysis system (Fig. 1). The median SLV of 24 patients was 1471.4 ml. The median FLR increased from 362 (range: 254–490) ml before first stage procedure to 615 ml before second stage procedure, and the median FLR/SLV increased from 25.3% (range: 17.4–29.9%) to 42% (range: 28.9–55.6%). The median FLRW increased from 310 g before first stage procedure to 517 (range: 260–656) g before second stage procedure, and the median FLRW/BW increased from 0.46 to 0.76%. The median interval between the two stages was 16 (range: 9–24) days. The median absolute KGR was 18.1 ml/day, and the median relative KGR was 5.1% (range: 2–10.3%) (Table 2). In order to explore the cause or mechanism of the rapid growth of FLR, we analyzed the remnant liver specimens sampled during two stages procedures. The results showed that the expression of YAP and p-JNK in liver remnant tissue of second stage procedure was significantly higher than that in liver remnant tissue of first stage procedure, and co-expression of YAP and p-JNK were observed (Fig. 2). In addition, the liver reserve function indicators, including alanine aminotransferase (ALT), aspartate aminotransferase (AST), international normal ratio (INR), ALB, PT, total bilirubin (TB) after 2 stage procedures are showed in Fig. 3.

**Table 2:** Pre- and post-stage 1 operative increases of FLR

Variable	Data, median (range)
SLV, ml	1471.4 (967.1–1980.9)
S1 preoperative	
FLR, ml	362 (254–490)
FLR/SLV (%)	25.3 (17.4–29.9)
FLRW, g	310 (213–412)
FLRW/BW (%)	0.46 (0.30–0.56)
S2 preoperative	
FLR, ml	615 (309–781)
FLR/SLV (%)	42.0 (28.9–55.6)
FLRW, g	517 (260–656)
FLRW/BW (%)	0.76 (0.52–1.02)
Interval time, day	16 (9–24)
Absolute KGR, ml/day	18.1 (5.8–37.7)
Relative KGR, %/day	5.1 (2–10.3)

SLV, standard liver volume; FLR, future liver remnant; FLRW, future residual liver weight; BW, body weight; KGR, kinetic growth rate

### Operation information

The surgical success rate of ALPPS was 91.7%. Of the 24 patients, 22 patients successfully completed two stage procedures, and 2 patients failed to undergo second stage procedure due to insufficient FLR. 7 patients underwent complete laparoscopic surgery, and 8 patients underwent open surgery, and 2 patients underwent conversion from laparoscopic to open procedure due to insufficient abdominal space during second stage procedure. The intraoperative blood loss in the laparoscopic procedure group was significantly less than that in the open procedure group. The time of postoperative anal exhaust and the interval time between two stages procedures were also shorter in the laparoscopic surgery group than those in the open procedure group. The indicator of ICG15 was significantly lower in the laparoscopic procedure group than that in the open procedure group on 7 d after first stage procedure of ALPPS (Table 3).

**Table 3:** Operation information

Group (S1^*^)	S1 blood loss (ml)	S1 Operative time (min)	S1 anal exhaust time (Day)	Interval time (Day)	S1 ICG15 (POD7)
Open (n = 8)	383.8 ± 56.19	287.1 ± 20.7	4.8 ± 0.45	18.8 ± 1.3	12.6 ± 2.5
Laparoscopic (n = 14)	235.7 ± 28.91	325.5 ± 33.54	3.4 ± 0.31	14.21 ± 1.0	8.0 ± 0.7
p values	0.02	0.31	0.02	0.01	0.04

ICG15, 15-min retention rate (R15) of indocyanine green; POD, post-operative day

^*^S1, the first stage procedure

### Prognosis

#### Postoperative complications

The main postoperative complications of ALPPS were pulmonary infection, abdominal infection, gastrointestinal bleeding, incision infection and bile leakage. The total postoperative complication incidence after first and second stage procedure was 54.5%, among which 2 cases of biliary leakage were above grade III complications (9.1%). After the first stage procedure, the incidence of complications in the open surgery group was 37.5%, which was significantly higher than that in the laparoscopic surgery group (14.3%). 2 cases of bile leakage above grade III complications occurred in the open surgery group. After second stage procedure, the incidence of complications in the open surgery group was 40%, which was also higher than that in the laparoscopic surgery group (20%) (Table 4).

**Table 4:** Postoperative complications of different methods of ALPPS

Group	Pulmonary infection (including pleural effusion)	Gastrointestinal bleeding	Abdominal infection	Incision infection	Bile leakage	Overall incidence
S1^*^						
Open (n = 8)	0 (0.0)	0 (0.0)	1 (12.5%)	0 (0.0)	2 (25%)	3 (37.5%)
Laparoscopic (n = 14)	1 (7.1%)	0 (0.0)	0 (0.0)	1 (7.1%)	0 (0.0)	2 (14.3%)
S2^#^						
Open (n = 15)	3 (20.0%)	0 (0.0)	2 (13.3%)	1 (6.7%)	0 (0.0)	6 (40%)
Laparoscopic (n = 5)	0 (0.0)	1 (20%)	0 (0.0)	0 (0.0)	0 (0.0)	1 (20%)

^*^S1, the first stage procedure

^#^S2, the second stage procedure

Among 330 patients with HBV-associated HCC treated with TACE, 22 patients were screened out by using PSM (1:1) to match as closely as possible the 22 patients who underwent ALPPS (Of the 24 patients treated with ALPPS, 2 were excluded because they did not undergo second stage procedure). The matching conditions and postoperative complications after matching were shown in Additional file 2: Table S1. In the TACE group, the main complications were gastrointestinal symptoms (nausea, vomiting and diarrhea), abdominal pain, fever and myelosuppression. The two groups had the same complication rate, but there were lower rate of above grade III complications in the TACE group than that in the ALPPS group (Table 5).

**Table 5:** Postoperative complications of ALPPS and TACE

Complications	< 3b	≥ 3b	Total
ALPPS (n = 22)			
Pulmonary infection	5	0	5
Gastrointestinal bleeding	1	0	1
Abdominal infection	2	0	2
Incision infection	2	0	2
Bile leakage	0	2	2
	10 (45.4%)	2 (9.1%)	12 (54.5%)
TACE (n = 22)			
Gastrointestinal symptom	5	0	5
Fever	4	0	4
Myelosuppression	1	0	1
Gastrointestinal bleeding	1	0	1
Hepatalgia	1	0	1
	12 (54.5%)	0 (0)	12 (54.5%)

#### Survival

Among the 24 patients treated with ALPPS, 1 patient died of multiple-organ failure within 90 days, and 2 patients did not undergo second stage procedure. The 1-year, 2-year and 5-year OS rates were 71.4%, 33.3% and 4.8% respectively, and the DFS rates were 57.1%, 14.3% and 0. Univariate analysis showed that operation methods (stage 1), interval time, tumor size, vascular invasion, tumor thrombus, ascites, AFP and portal hypertension were significant predictors of OS, and operation method (stage 1), interval time, tumor size, vascular invasion, tumor thrombus, AFP and portal hypertension were significant predictors of DFS. The interval time was independent risk factors for both OS and DFS (Additional file 2: Table S2).

**Fig. 4:**
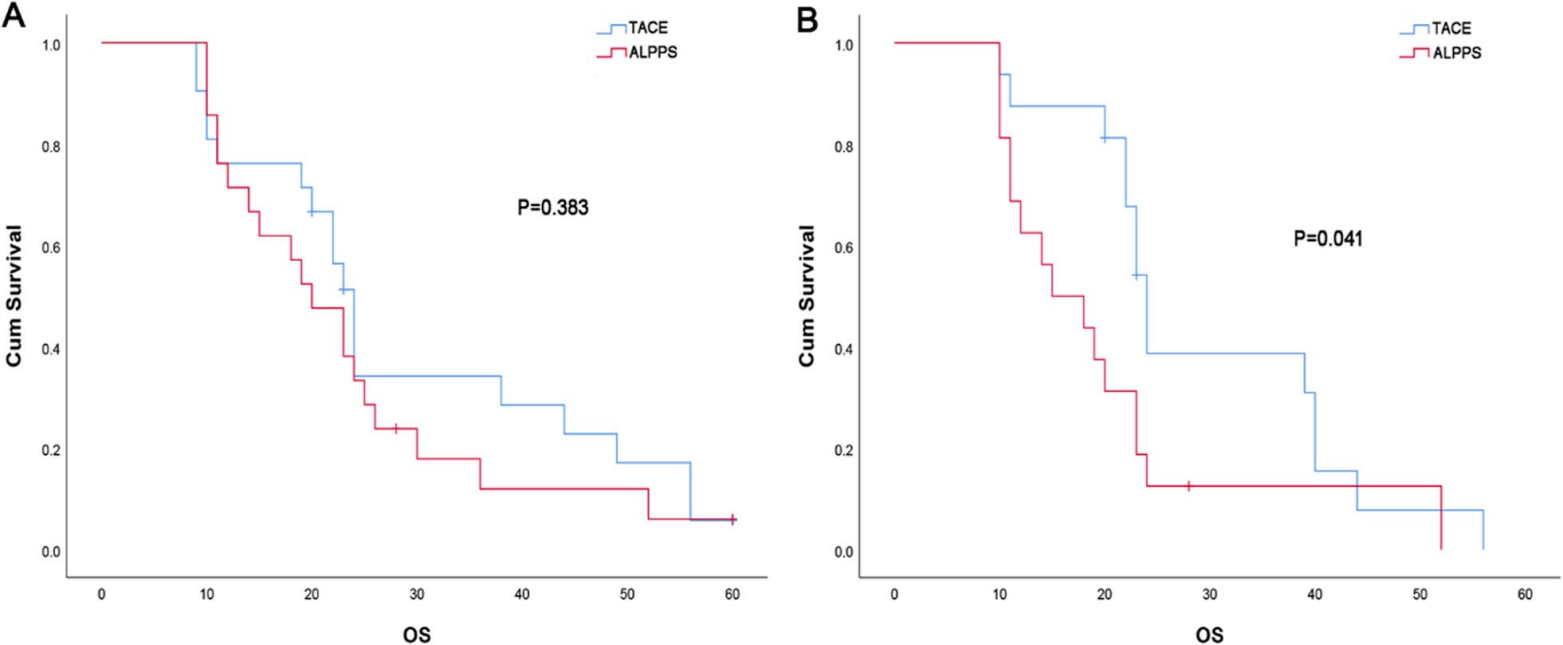
The OS rates of HBV- associated HCC patients using PSM (1:1) comparative analyses of ALPPS with TACE. **A** There was no statistical difference in OS rates of HBV-associated HCC patients between ALPPS and TACE (n = 21, p = 0.383); **B** There was a significant difference in OS rates of HBV-associated HCC patients (BCLC stage B and C between ALPPS and TACE (n = 16, p = 0.041)

A 1:1 PSM was done to select 21 patients from the 330 patients treated by TACE to match as closely as possible the 21 patients who underwent ALPPS. The matching conditions and postoperative survival analysis after matching were shown in Additional file 2: Table S3. The 1-year, 2-year and 5-year OS rates of the TACE group were 76.2%, 38.1%, and 4.8%. There was no significant difference in OS between the ALPPS group and TACE group (Fig. 4A).

PSM was also used to match 16 BCLC stage B and C patients treated with TACE to 16 BCLC stage B and C patients with ALPPS. The matching conditions and postoperative survival analysis after matching were shown in Additional file 2: Table S4. The 1-year, 2-year and 5-year OS rates were 62.5%, 12.5%, 0 in the ALPPS group, and 87.5%, 43.8%, 0 in the TACE group. The OS rates in the ALPPS group were lower than those in the TACE group (Fig. 4B). Interestingly, for matched 16 patients undergoing ALPPS, further PSM analysis revealed that patients undergoing L-ALPPS had no significant difference in OS rates compared with those treated with, and patients undergoing open procedure had significantly lower OS than patients undergoing TACE (Additional file 1: Fig S1).

## Discussion

HCC is one of the most common malignancies, with the fifth highest morbidity and the third highest mortality rate [21]. Liver transplantation is the optimal treatment for HCC, which is too difficult to achieve due to the shortage of donor livers. Clinically, one-step radical hepatectomy is the first-line treatment. However, the patient’s poor general condition, the poor hepatic function and the insufficient FLR, that is unresectable HCC, makes the radical hepatectomy impossible. TACE seems to be the most suitable method for initially unresectable HCC, but there are some complications after TACE, such as liver failure, liver abscess, hepatic encephalopathy and other serious complications. Additionally, there is still some doubt about the long-term efficacy of TACE. With the development of surgical materials and techniques, staged hepatectomy based on portal venous ligation and portal venous embolization is gradually applied for the treatment of initially unresectable HCC. But it has been criticized due to the long waiting interval that may leads to progression of the tumor. ALPPS was first introduced by de Santibañes et al. in 2012 [6] and has been gradually accepted by surgeons for its ability to rapidly increase the FLR in a short period of time, but it is still controversial for its high early complication and mortality rate [22, 23].

Currently, ALPPS is mainly applied in patients with liver metastasis from colorectal cancer, rarely in HCC patients. Schadde et al. believed [24] that HCC itself was an independent risk factor for ALPPS. D’Haese et al. found [25] that the 90-day mortality of HCC patients treated with ALPPS was significantly higher than that of patients with liver metastasis from colorectal cancer treated with ALPPS (31% vs. 7%, *p* = 0.001). Chan et al. found [26] that there was no significant difference in 90-day mortality between ALPPS and PVE (6.5% vs. 5.8%) in their study comparing short-term survival between ALPPS and PVE. Wang et al. found [27] that the 90-day mortality rate of ALPPS patients after surgery was 11.1%. By comparing the long-term survival rates, they found that ALPPS was significantly better than TACE. In our study, the 90-day mortality of 22 patients treated with ALPPS was 4.5%. PSM analysis showed no significant difference in OS between patients treated with ALPPS and TACE. Notably, the univariate analysis found that method of first stage procedure is a significant risk factor for OS. Additionally, compared with open ALPPS, L-ALPPS had shorter interval time between the two stage procedures and could avoid the progress of tumor. Moreover, laparoscopy procedure is more in line with the “no touch” technique, which reduces the risk of tumor implantation and metastasis to a certain extent, and is more in line with the “damage control surgery” paradigm. All the same, the speculation that L-ALPPS can significantly improve the OS need to be verified by large-scale analyses.

Limitations of ALPPS include its complications, mortality and unsatisfactory growth of FLR, which may lead to tumor progression and loss of radical resection opportunities for some patients. In addition, the effect of ALPPS on tumor recurrence and metastasis is still unclear. Once the ALPPS operation fails, the only alternative will be liver transplantation or palliative treatment, such as interventional and targeted therapy, immunotherapy. Postoperative complications of ALPPS mainly include biliary leakage, pleural and peritoneal effusion, infection, acute liver failure and so on [28, 29]. A retrospective study of 46 patients treated with ALPPS by Chan et al. showed [26] that the total incidence of postoperative complications was 20.7%, with 21.7% for first stage procedure and 19.6% for second stage procedure. Another retrospective study of 45 patients treated with ALPPS reported by Wang et al. showed [27] that the incidence of postoperative complications was 37.8% in first stage procedure and 56.1% in second stage procedure, and the incidence of above grade III postoperative complications was 11.1%. A systematic study reported by Zhang et al. showed [30] that the incidence of above grade III postoperative complications of ALPPS were 0–25% in first stage procedure and 0–45% in second stage procedure. In our study, the total postoperative complications of ALPPS were 54.5%, with 22.7% in first stage procedure and 35% in second stage procedure. The incidence of above grade III postoperative complications of ALPPS was 9.1%, which was slightly lower than that in the study reported by Wang et al. It is worth noting that the postoperative complications of L-ALPPS were less than those of open surgery. These data fully showed that although there are many postoperative complications of ALPPS in the early stage, with the improvement of technology and methods, ALPPS became more and more safe. In recent years, radiofrequency-assisted ALPPS [31], partial ALPPS [32], and L-ALPPS [33] have emerged for the treatment of HCC. Especially for L-ALPPS, there was less injury to bile and important blood vessels in the process of portal vein ligation and liver parenchymal dissection because laparoscopy can provide a better visual field and reduce intraoperative turnover, etc.

Since it was reported in 2012, ALPPS has attracted extensive attention because it can promote rapid growth of the FLR in a short interval. ALPPS is applied in HCC patients with insufficient FLR to obtain the opportunity to remove secondary liver tumors. Ke et al. found [34] that the average growth rate of FLR was 50%, and the average interval between first and second stage procedures was 12 days, and the completion rate of two stage procedures was 87% in 23 cases of ALPPS. Wang et al. found [27] that the average growth rate of FLR was 56.8%, and the average interval was 12 days, and the completion rate of two-stage procedures was 91.1% in 45 cases of ALPPS. In our study, among 21 patients treated with ALPPS, the average growth rate of FLR was 58.9%, and the average interval was 16 days, and the completion rate of two-stage procedure was 91.7%. The above data have shown that FLR increased rapidly in a short interval in procedure of ALPPS, but the mechanism of rapid FLR increase is not clear. Some studies have suggested [35, 36] that the rapid increase in FLR over a short period of time is the result of hypertrophy. However, some researchers found [37, 38] that the increase in FLR was the result of effective proliferation of liver cells, which was not caused by steatosis or edema. Most scholars believe [39–41] that the mechanisms of FLR increase mainly include as follow: (1) Hepatocyte regeneration is related to portal vein and hepatic artery blood flow. Hepatic blood flow is redistributed after surgery, and portal vein blood flow completely flowed into the remaining liver, resulting in hepatocyte regeneration. (2) The diseased liver still has arterial blood flow. On the one hand, the diseased liver can regulate hemodynamics that avoids portal hypertension in the remaining liver. On the other hand, the diseased liver can participate in liver synthesis, metabolism and detoxification as an “accessory liver”, creating favorable conditions for the growth of FLR. (3) The surgery creates an inflammatory and hypoxic environment. On the one hand, macrophages and other immune cells rapidly differentiate and participate in wound repair and hepatocyte regeneration. On the other hand, HGF and other humoral factors promote the proliferation of hepatocytes. Additionally, the activation of the VEGF pathway and proliferation of sinusoidal endothelial cells can reconstruct the normal structure of hepatic sinusoids and contribute to liver regeneration. Langiewicz et al. found [40] that the expression of the mitogen-activated protein kinase 8 (or JNK1) was higher than that of the control group by analyzing the gene expression profile of liver differentiation in mice treated with ALPPS. After first stage procedure of ALPPS, activated JNK1 promoted the expression of GLI1, Cyclin D1 and other proliferation markers. However, the expression of GLI1 and Cyclin D1 decreased in the mice treated with JNK inhibitors, and liver cell regeneration slowed down. The Hippo pathway is a cascade of enzymatic reactions. When the Hippo pathway is activated, MST1/2 phosphorylates salvador 1, which facilitates the MST1/2-LATS1/2 interaction and then phosphorylates YAP. Phosphorylated Yap is degraded in the cytoplasm [10]. Recent studies have shown [8, 42] that activation of YAP is critical for liver tissue repair and regeneration after hepatectomy. Our study found that the expression of YAP and p-JNK in liver remnant tissue of stage 2 procedure was significantly higher than that in liver remnant tissue of stage 1 procedure, and co-expression of YAP and p-JNK was observed. Our results indicated that Hippo signaling and JNK signaling pathway might affect hepatocyte regeneration after ALPPS, but the specific regulatory mechanism is unclear and further experimental verification is needed.

ALPPS is a promising procedure for treating initially unresectable primary hepatocellular carcinoma, especially the application of laparoscopy, which can significantly reduce the incidence of complications and might improve the effect of ALPPS. ALPPS is safe and feasible procedure in terms of short-term effect, but its long-term effect needs to be studied. Hippo signaling and JNK signaling pathway might mediating the rapid increase of FLR in ALPPS procedure. The mechanism of rapid FLR increase in ALPPS procedure remains further investigated.

## Conclusions

ALPPS is a safe and effective treatment for initially unresectable HBV-associated HCC. Laparoscopic technique might improve the effect of ALPPS. YAP and JNK pathway might take a role in rapid FLR increase in ALPPS procedure.

## Supplementary Information

**Additional file 1**. Supplementary Figure.

**Additional file 2.** Supplementary Table.

## Data Availability

The datasets used and/or analysed during the current study are available from the corresponding author on reasonable request.

## Abbreviations

ALPPS: Associating liver partition and portal vein ligation for staged hepatectomy
HBV: Hepatitis B virus
HCC: Hepatocellular carcinoma
FLR: Future liver remnant
PSM: Propensity score matching
TACE: Transarterial chemoembolization
SLV: Standard liver volume
L-ALPPS: Laparoscopic-ALPPS
OS: Overall survival
YAP: Yes-associated protein
MRI: Magnetic resonance imaging
CT: Computed tomography
BCLC: Barcelona Clinical Liver Cancer Staging System
SLV: Standard liver volume
FRLW: Future residual liver weight
KGR: Kinetic growth rate
PT: Prothrombin time
ICG: Indocyanine green
NRS: Nutrition risk screening
ALB: Albumin
ALT: Alanine aminotransferase
AST: Aspartate aminotransferase
INR: International normal ratio
TB: Total bilirubin
