# Supplementary material for: Application of associating liver partition and portal vein ligation for staged hepatectomy for initially unresectable hepatocellular carcinoma
Source: BMC Surg. 2022 Nov 24;22:407. doi: 10.1186/s12893-022-01848-w (PMC9700990; doi:10.1186/s12893-022-01848-w)
Supplement: Supplementary file 1 — Additional file 1. Supplementary Figure. [file 12893_2022_1848_MOESM1_ESM.docx]

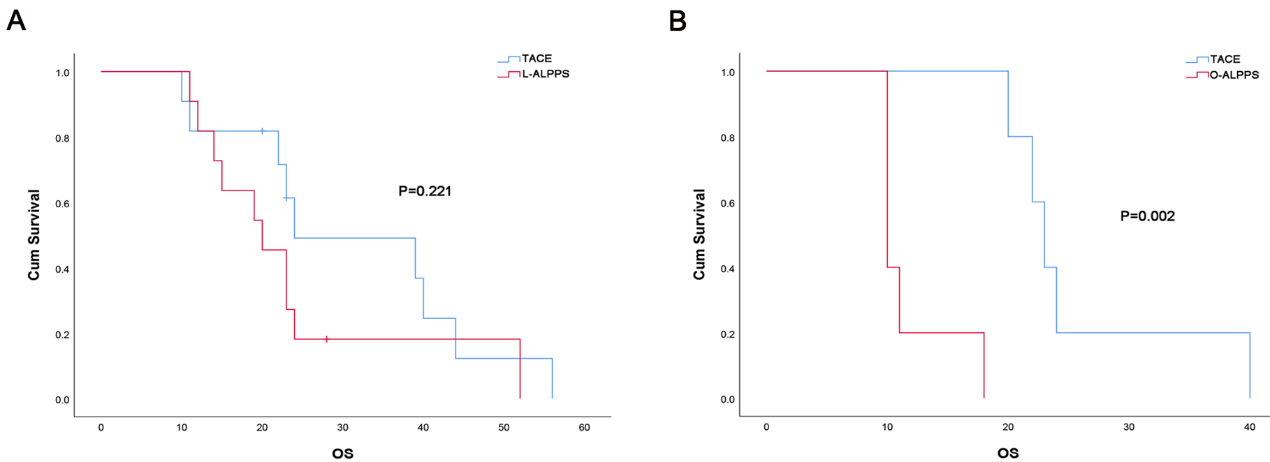


**Figure S1 The OS rates of HBV- associated HCC patients (BCLC stage B and C) using PSM (1:1) comparative analyses of ALPPS with TACE** (A) There was no statistical difference in OS rates of HBV-associated HCC patients between L-ALPPS and TACE (n=10, P=0.221); (B) There was a significant difference in OS rates of HBV- associated HCC patients between O-ALPPS and TACE (n=6, P=0.002); L-ALPPS, Laparoscopic-ALPPS; O-ALPPS, Open-ALPPS.


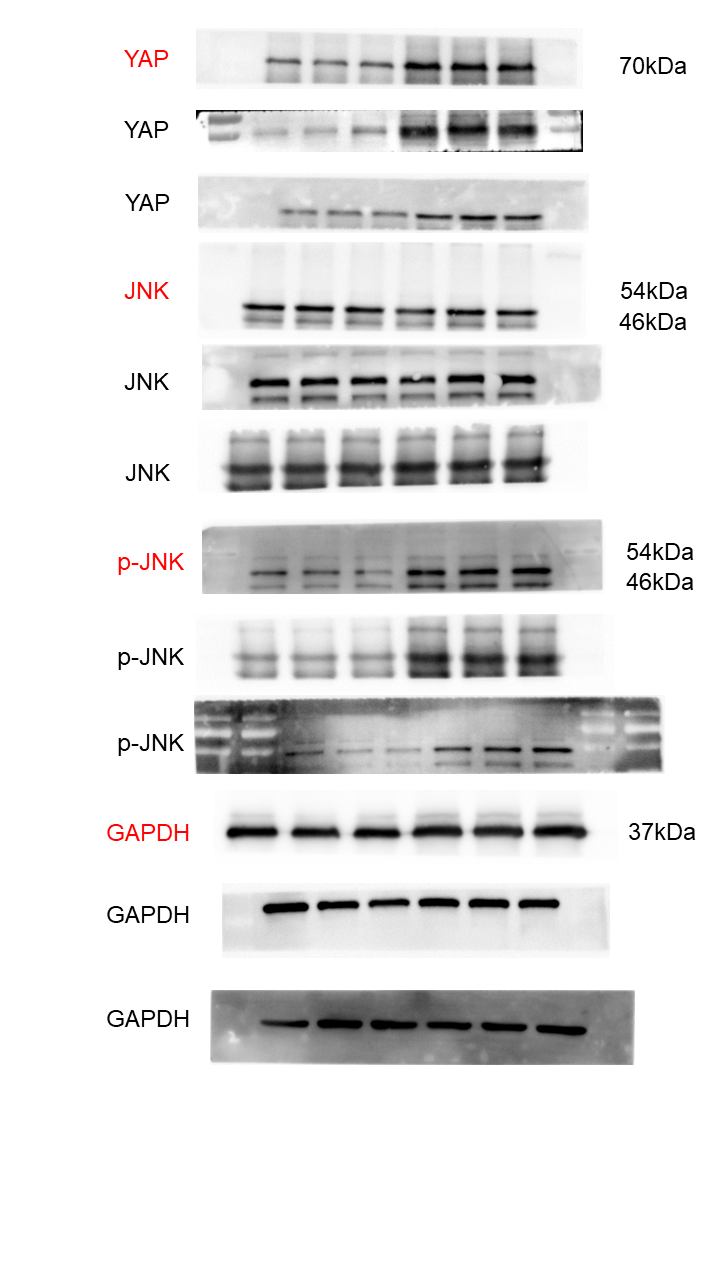


**Figure S2 Original blots of YAP, JNK, p-JNK and GAPDH.** After the successful transfer of protein on PVDF membranes, membranes were blocked and eventually cropped horizontally according to the desired protein of interest. Thereafter each membrane was processed for respective antibody incubation and detection. Red represented the images to the figure 2A in the manuscript.
