# Supplementary material for: Application of associating liver partition and portal vein ligation for staged hepatectomy for initially unresectable hepatocellular carcinoma
Source: BMC Surg. 2022 Nov 24;22:407. doi: 10.1186/s12893-022-01848-w (PMC9700990; doi:10.1186/s12893-022-01848-w)
Supplement: Supplementary file 2 — Additional file 2. Supplementary Table. [file 12893_2022_1848_MOESM2_ESM.docx]

**Table S1** Comparisons of matching conditions for postoperative complications of ALPPS (PSM,1:1, n=22)

|  | Before PSM (Original Cohort) | | | After PSM (Matched Cohort) | | |
| --- | --- | --- | --- | --- | --- | --- |
|  | ALPPS | TACE | *P* | ALPPS | TACE | *P* |
|  | (n=22) | (n=330) |  | (n=22) | (n=22) |  |
| Age (years) | 49.55±11.75 | 55.30±13.41 | 0.500 | 49.55±11.75 | 49.05±17.26 | 0.911 |
| Sex (Male/Female) | 22/0 | 297/33 | 0.238 | 22/0 | 22/0 | - |
| NRS* score (2/3/4) | 16/5/1 | 177/132/21 | 0.122 | 16/5/1 | 12/9/1 | 0.307 |
| MELD^#^ score | 7.44±1.46 | 5.99±3.85 | <0.001 | 7.44±1.46 | 6.75±3.45 | 0.400 |
| AFP^ (ng/ml) | 4280±10211 | 2221±6189 | 0.360 | 4280±10211 | 3616±9986 | 0.828 |
| Tumor number |  |  |  |  |  |  |
| 1 | 14 | 184 | 0.471 | 14 | 14 | - |
| >1 | 8 | 146 |  | 8 | 8 |  |
| Tumor size (cm) | 7.37±3.66 | 7.16±4.73 | 0.841 | 7.37±3.66 | 8.53±4.92 | 0.379 |
| Macroscopic vascular invasion |  |  |  |  |  |  |
| No | 16 | 252 | 0.698 | 16 | 17 | 0.728 |
| Yes | 6 | 78 |  | 6 | 5 |  |
| Distant metastasis |  |  |  |  |  |  |
| No | 22 | 292 | 0.183 | 22 | 22 | - |
| Yes | 0 | 38 |  | 0 | 0 |  |
| Lymphatic node metastasis |  |  |  |  |  |  |
| No | 22 | 288 | 0.149 | 22 | 22 | - |
| Yes | 0 | 42 |  | 0 | 0 |  |
| Ascites |  |  |  |  |  |  |
| None | 11 | 244 | 0.019 | 11 | 14 | 0.475 |
| Low | 9 | 80 |  | 9 | 8 |  |
| Medium | 2 | 6 |  | 2 | 0 |  |
| ALB^&^(g/L) | 38.80±5.75 | 40.26±7.66 | 0.381 | 38.80±5.75 | 37.26±7.17 | 0.434 |

^*^NRS, Nutritional risk screening; ^#^MELD, The Model of End-Stage Liver Disease score; ^^^AFP, alpha fetoprotein; ^&^ALB, albumin.

**Table S2** Variables analysis of risk factors for prognosis after ALPPS: (A) Univariate analysis of risk factors for prognosis (OS and DFS) after ALPPS; (B) Independent risk factors for both OS; (C) Independent risk factors for both DFS. AST, aspartate aminotransferase; ALT, alanine aminotransferase; ALB, albumin; TB, total bilirubin; AFP, alpha fetoprotein; ICG15, 15-minute retention rate (R15) of indocyanine green; S1, the first stage procedure; S2, the second stage procedure.


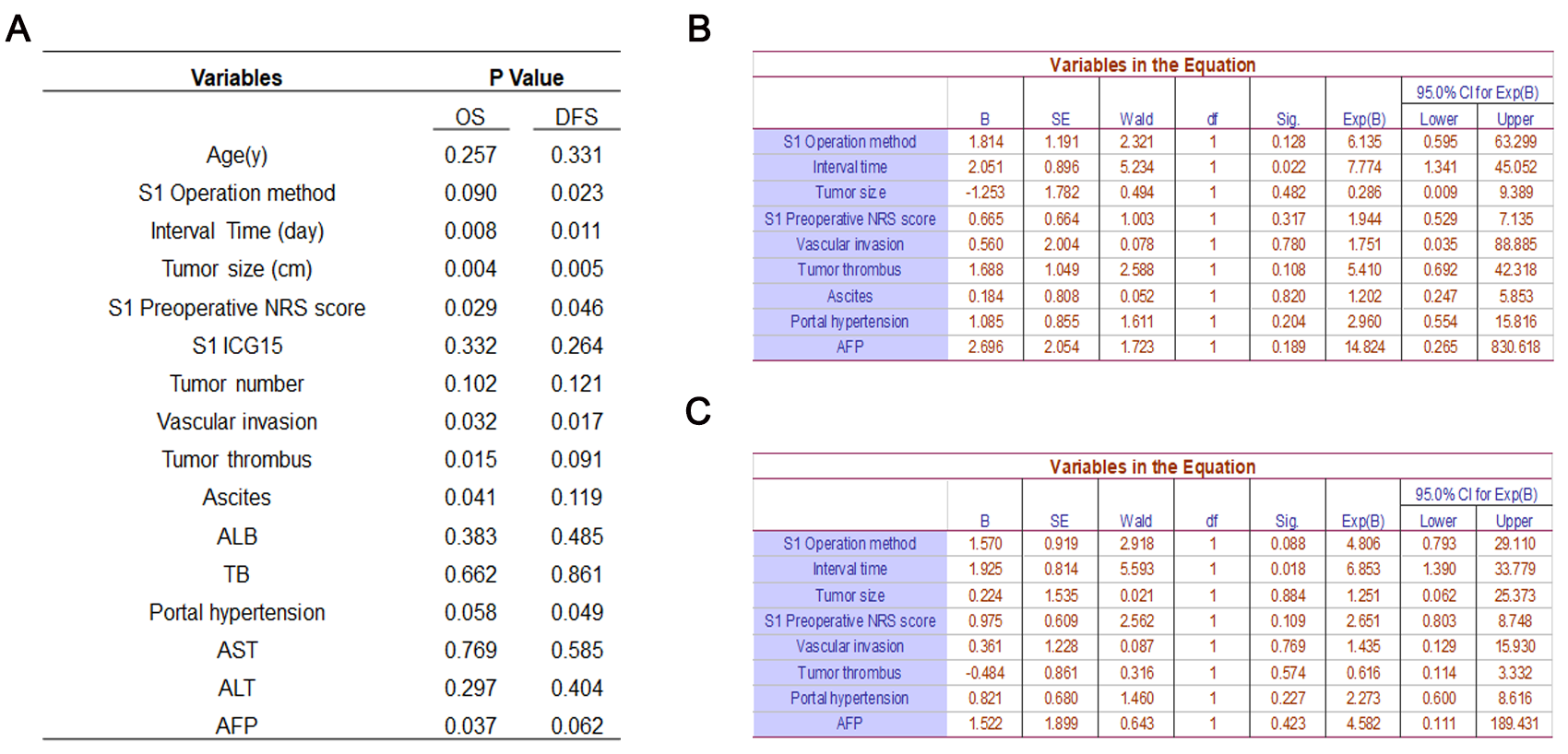


**Table S3** Comparisons of matching conditions for prognosis (OS and DFS) after ALPPS (PSM, 1:1, n=21)

|  | Before PSM (Original Cohort) | | | After PSM (Matched Cohort) | | |
| --- | --- | --- | --- | --- | --- | --- |
|  | ALPPS | TACE | *P* | ALPPS | TACE | *P* |
|  | (n=21) | (n=330) |  | (n=21) | (n=21) |  |
| Age (years) | 48.24±10.28 | 55.30±13.41 | 0.018 | 48.24±10.28 | 46.90±13.08 | 0.715 |
| Sex (Male/Female) | 21/0 | 297/33 | 0.241 | 21/0 | 20/1 | 1.000 |
| NRS* score (2/3/4) | 16/4/1 | 177/132/21 | 0.118 | 16/4/1 | 14/7/0 | 0.770 |
| MELD^#^ score | 7.42±1.49 | 5.99±3.84 | 0.091 | 7.42±1.49 | 6.95(1.32-15.5) | 0.484 |
| AFP^ (ng/ml) | 3354±9469 | 2221±6189 | 0.434 | 3354±9469 | 3542±10127 | 0.951 |
| Tumor number |  |  |  |  |  |  |
| 1 | 14 | 184 | 0.372 | 14 | 12 | 0.751 |
| >1 | 7 | 146 |  | 7 | 9 |  |
| Tumor size (cm) | 7.26±3.71 | 7.16±4.73 | 0.928 | 7.26±3.71 | 7.84±4.92 | 0.668 |
| Macroscopic vascular invasion |  |  |  |  |  |  |
| No | 16 | 252 | 1.000 | 16 | 18 | 0.697 |
| Yes | 5 | 78 |  | 5 | 3 |  |
| Distant metastasis |  |  |  |  |  |  |
| No | 21 | 292 | 0.199 | 21 | 20 | 1.000 |
| Yes | 0 | 38 |  | 0 | 1 |  |
| Lymphatic node metastasis |  |  |  |  |  |  |
| No | 21 | 288 | 0.163 | 21 | 21 | 1.000 |
| Yes | 0 | 42 |  | 0 | 0 |  |
| Ascites |  |  |  |  |  |  |
| None | 11 | 244 | 0.026 | 11 | 14 | 0.627 |
| Low | 8 | 80 |  | 8 | 6 |  |
| Medium | 2 | 6 |  | 2 | 1 |  |
| ALB^&^(g/L) | 38.70±5.87 | 40.30±7.66 | 0.361 | 38.70±5.87 | 36.91±6.91 | 0.372 |

**Table S4** Comparisons of matching conditions for prognosis (OS and DFS, BCLC B+C) after ALPPS (PSM, 1:1, n=16)

|  | Before PSM (Original Cohort) | | | After PSM (Matched Cohort) | | |
| --- | --- | --- | --- | --- | --- | --- |
|  | ALPPS | TACE | *P* | ALPPS | TACE | *P* |
|  | (n=16) | (n=252) |  | (n=16) | (n=16) |  |
| Age (years) | 48.94±9.96 | 55.60±13.19 | 0.048 | 48.94±9.96 | 47.56±16.30 | 0.776 |
| Sex (Male/Female) | 16/0 | 228/24 | 0.400 | 16/0 | 16/0 | - |
| NRS* score (2/3/4) | 11/4/1 | 135/104/13 | 0.389 | 11/4/1 | 9/7/0 | 0.458 |
| MELD^#^ score | 7.57±1.28 | 6.53±3.95 | 0.014 | 7.57±1.28 | 7.14±2.40 | 0.530 |
| AFP^ (ng/ml) | 4400±10706 | 2685±6381 | 0.322 | 4400±10706 | 2192±2768 | 0.431 |
| Tumor number |  |  |  |  |  |  |
| 1 | 14 | 184 | 0.324 | 14 | 13 | 1.000 |
| >1 | 2 | 68 |  | 2 | 3 |  |
| Tumor size (cm) | 8.69±3.03 | 8.68±4.42 | 0.994 | 8.69±3.03 | 9.53±4.31 | 0.527 |
| Macroscopic vascular invasion |  |  |  |  |  |  |
| No | 11 | 174 | 1.000 | 11 | 11 | - |
| Yes | 5 | 78 |  | 5 | 5 |  |
| Distant metastasis |  |  |  |  |  |  |
| No | 16 | 214 | 0.191 | 16 | 16 | - |
| Yes | 0 | 38 |  | 0 | 0 |  |
| Lymphatic node metastasis |  |  |  |  |  |  |
| No | 16 | 210 | 0.155 | 16 | 16 | - |
| Yes | 0 | 42 |  | 0 | 0 |  |
| Ascites |  |  |  |  |  |  |
| None | 7 | 179 | 0.011 | 7 | 7 | 0.624 |
| Low | 7 | 69 |  | 7 | 9 |  |
| Medium | 2 | 4 |  | 2 | 0 |  |
| ALB^&^(g/L) | 38.76±6.30 | 39.93±7.86 | 0.558 | 38.76±6.30 | 37.39±8.34 | 0.606 |
